# 

**Published:** 1848

**Authors:** 


### NOTICE TO READERS AND CORRESPONDENTS.

Our patrons, we hope, will recollect that our terms are payment in advance. Those who are in arrears will confer a favour by remitting the amount due. If any subscribers wish to discontinue the Journal they must do so at the end of a volume.

To receive attention letters must come post paid. We have heretofore received a great many in reference to changing the direction of the Journal and other matters of no pecuniary interest to us, upon which the postage was not paid.

We have received the following original communications:

Sanguinaria Canadensis as a Therapeutical agent. By J. L. Mothershead, M. D.

Quinine. By E. C. Banks, M. D.

Post mortem examination of a case of abscess of the liver. By H. Rosenkrans, M. D.

A case of strangulated Inguinal Hernia, mistaken for Bilious Cholic and treated accordingly by a Thomsonian. Reported by R. Hoisley, Medical Student.

Diseases of the West—their complications &c. By W. Matthews, M. D., of Eberle, Ind.

Remarks upon Puerperal Fever complicated with Malaria. By E. R. Parks, M. D. Also, from the same author, Cases of Malignant Pneumonia, with remarks.

Iodine an antidote to the bite of the Rattle-snake. B. Jas. S. Whitmire, M. D.

We have also received from the publishers the following works, which are for sale by Jos. Keene, Jr. & Bro., Chicago:

A Dispensatory or Commentary on the Pharmacopœas of Great Britain (and the United States) etc. etc. By Robert Christoson, M. D., V. P. R. S. E. &c. &c. Second Edition Revised and Improved, etc. etc. With 213 illustrations. Edited by R. Eaglesfield Griffith, M. D., etc. From Lea & Blanchard. pp. 1008.

The principles and pratice of Modern Surgery. By Robert Druitt, F. R. C. S. Edited by F. W. Sargent, M. D., etc, etc., illustrated with 193 wood engravings. Lea & Blanchard. 1848. pp. 576.

A Dictionary of Medical Science, containing a concise explanation of the various subjects and terms; with the French and other synonymes: notices of climate and celebrated mineral waters: formula for various officinal and empyrical preparations, etc.. Seventh edition, carefully revised and greatly enlarged. Lea & Blanchard: Philadelphia. 1848. pp. 912.

Females and their Diseases. A series of letters to his class. By Charles D. Meigs, M. D. Lea & Blanchard. 1848. pp. 670.

Principles of Medicine. Comprising general pathology and Therapeutics, and a brief general view of Etiology, Nosology, Semiology, Diagnosis, Prognosis, and Hygienics. By Charles J. B. Williams, M. D., F. R. S., etc. etc. Edited with additions, by Meredith Clymer, M. D., etc. etc., third American from the second Enlarged London Edition. Lea & Blanchard. 1848. pp. 440.

An account of some of the most important diseases peculiar to Women. By Robert Gooch, M. D. With Illustrations: Second Edition. Philadelphia Ed. Barryton & Geo D. Haswell. 1848. pp. 322.

A System of Clinical Medicine. By Robert James Graves, M. D., M. R. I. A., etc. etc. With Notes and a series of Lectures, by W. W. Gerhard, M. D., &c. &c., third American Edition. Philadelphia Ed. Barryton & Geo. D. Haswell. 1848. pp. 750.

Gardner's Medical Chemistry and Wilson's Anatomy, from Lea & Blanchard. Dufton on the Ear, Churchill's Midwifery, and Bartlett on Certainty in Medicine. Also our usual Exchange list.

### RECEIPTS FOR THIS JOURNAL FOR SEPTEMBER AND OCTOBER, 1848.

### INDIANA.

| | |
|---|---|
| Dr. B. F. Russell, - - - | $2 00 |
| Dr. Frost, - - - - - - | 2 00 |
| Dr. Latta Wallace, - - - - | 2 00 |
| Dr. D. T. Yeakel, - - - | 2 00 |
| Dr. Clark, - - - - - - | 2 15 |
| Drs. Bamford & Treadway, - - | 2 00 |
| Dr. J. H. Buell, - - - - | 4 00 |
| Dr. W. T. Green, - - - - | 2 00 |
| Dr. A. L. Pettijohn, - - - - | 2 00 |
| Dr. D. D. Hall, - - - - | 2 00 |
| Dr. S. V. Miller, - - - - | 2 00 |
| Dr. C. H. Butler, - - - - | 1 00 |
| Dr. T. Bullard, - - - - - | 2 00 |
| Dr. R. S. Robinson, - - - | 2 00 |
| Dr. B. Bartholomew, - - - - | 2 00 |
| Dr. W. H. Davis, - - - | 2 00 |
| Dr. W. P. Demmitt, - - - | 2 00 |
| Dr. W. Matthews, - - - | 2 00 |
| Dr. A. P. Mendenhall, - - - | 1 00 |
| Dr. J. W. P. Sellars, - - - | 2 00 |
| Dr. R. Trowbridge, - - - - | 2 00 |
| Dr. W. Mahan, - - - - | 2 00 |
| Dr. G. W. Cleppinger, - - - | 2 00 |
| Dr. W. C. Hopwood, - - - | 2 00 |
| Dr. J. J. Shryock, - - - - | 2 00 |
| Dr H. W. Mann, - - - - | 4 00 |
| Dr H. P. Howes, - - - - | 2 00 |
| Dr P. S. Cole, - - - - | 1 00 |
| Dr R. Willard, - - - - - | 2 00 |
| Dr Geo. Parks, - - - - | 2 00 |
| Dr S. J. Bowlby - - - - | 2 00 |
| Dr Wm. Parks, - - - - | 2 00 |
| Dr Jno. F. Parks, - - - - | 2 00 |
| Dr R. Brown, - - - - | 1 00 |
| “ S. Russell, - - - - - | 2 00 |
| “ A. G. Mandiford, - - - | 2 00 |
| “ A. Teegarden, - - - - | 2 00 |
| “ L. H. Sovereign, - - - | 2 00 |
| “ J. Bedsford, - - - - - | 1 00 |
| “ M. M. Latta, - - - - | 2 00 |
| “ P. Henkle, - - - - - | 2 00 |
| “ S. B. Kiler, - - - - | 2 00 |
| “ L. Barber, - - - - - | 1 00 |
| “ W. C. Clarke, - - : - | 2 00 |
| “ T. P. Bicknell, - - - - | 2 00 |
| “ McHugh, - - - - | 2 00 |
| “ E. Jones, - - - - - | 1 00 |
| “ Shelden, - - - - - | 3 95 |
| “ S. S. Thompson, - - - - | 2 00 |
| “ Walter Hay, - - - - | 1 25 |

### INDIANA.

| | |
|---|---|
| Dr. J. R. Coxe, - - - - | 2 00 |
| “ L. D. Hogle, - - - - | 2 00 |
| “ G. Bolles, - - - - - | 2 00 |
| “ S. Morris, - - - - | 1 00 |
| “ C. C. Robinson, - - - - | 2 00 |
| “ J. E. Lloyd, - - - - | 1 00 |

### OHIO.

| | |
|---|---|
| Dr. Patton, - - - - - | $2 00 |
| “ Morris, - - - - - | 1 00 |
| “ L. Gishwiller, - - - - | 1 00 |
| “ Roberts, - - - - - | 2 00 |
| “ O. H. Allen, - - - - | 2 00 |
| “ R. B. Cooper, - - - - | 1 00 |
| “ H. P. Wagner, - - - - | 2 00 |

### ILLINOIS.

| | |
|---|---|
| Dr. E. K. Farmer, - - - - | $3 00 |
| “ N. Wilson, - - - - | 2 00 |
| “ D. Ray, - - - - - - | 2 00 |
| “ A. S. Hudson, - - - | 2 00 |
| “ L. M. Logan, - - - - | 2 00 |
| “ E. L. Patton, - - - - | 2 00 |
| “ McGirr, - - - - - | 2 00 |
| “ L. Clark, - - - - | 2 00 |
| “ E. Sanford, - - - - - | 2 00 |
| “ J. W. Green, - - - - | 2 00 |
| “ G. W. Wentworth, - - - | 2 00 |
| “ J. V. Goltra, - - - - | 2 00 |
| “ C. H. Quinlan, - - - - | 2 00 |
| “ C. C. Barnes, - - - - | 2 00 |
| “ H. K. Hyslop, - - - - | 2 00 |
| “ A. W. Rawson, - - - | 2 00 |
| “ T. Wilkins, - - - - | 2 00 |
| “ Jos. M. Harnett, - - - | 1 0[illegible] |
| “ W. W. R. Woodbury. - - | 2 0[illegible] |

### MICHIGAN.

| | |
|---|---|
| Dr. A. Calkins, - - - - | $2 00 |
| “ J. P. Sawyer, - - - - | 3 00 |
| “ T. Upjohn, - - - - - | 2 00 |
| “ L. Foster, - - - - | 2 00 |
| “ J. S. M. C. Van Ness, - - | 2 00 |
| “ J. W. Phelps, - - - - | 2 00 |
| “ J. G. Cornell, - - - - | 2 00 |
| “ H. Knapp, - - - - | 2 00 |
| “ S. Russell, - - - - - | 2 00 |
| “ D. W. Smead, - - - - | 2 00 |
| “ John W. Hull. - - - - | 1 00 |

### WISCONSIN.

| | |
|---|---|
| Dr. D. Tuttle. - - - - | $1 00 |

### NORTH-WESTERN
### MEDICAL AND SURGICAL JOURNAL.

EDITED BY
W. B. HERRICK, M. D., AND JOHN EVANS, M. D.
PROFESSORS IN RUSH MEDICAL COLLEGE.

THE NORTH-WESTERN MEDICAL AND SURGICAL JOURNAL

Is issued simultaneously at Chicago, Illinois, and Indianapolis, Indiana, on the first of every other month, beginning with May, in numbers, each containing from 88 to 96 pages, neatly printed with new type and on good paper, and making, at the end of the year, a volume of about 550 pages, octavo, with title page and index complete.

Contributions to our pages, especially from physicians of the North-west, are earnestly solicited.

TERMS.—Two Dollars per annum, *in advance*, or Eight Dollars in one remittance for five copies, which may be sent by mail at our risk or paid to either of the Editors or authorized agents.

CHICAGO:
PRINTED BY J. W. DUZAN & Co.,
*Corner of Clark and Randolph Streets.*

